# Emerging insights into the biology of metastasis: A review article

**DOI:** 10.22038/ijbms.2019.32786.7839

**Published:** 2019-08

**Authors:** Soussan Irani

**Affiliations:** 1 Dental Research Centre, Oral Pathology Department, Dental Faculty, Hamadan University of Medical Sciences, Hamadan,Iran, Lecturer at Griffith University, Gold Coast, Australia

**Keywords:** Biomarkers, Circulating cells, Exosomes, Extracellular matrix, Metastasis, MicroRNAs, Neoplasms, Stem cells

## Abstract

Metastasis means the dissemination of the cancer cells from one organ to another which is not directly connected to the primary site. Metastasis has a crucial role in the prognosis of cancer patients. A few theories, different types of cell and several molecular pathways have been proposed to explain the mechanism of metastasis. In this work, the related articles in the limited period of time, 2000–mid -2018 were reviewed, through search in PubMed, Google Scholar and Scopus database. The articles published in the last two decades related to the biology of cancer metastasis were selected and the most important factors were discussed. Metastasis is critical factor to predict survival in patients with advanced cancer and prognosis determines the treatment plan. Many different cell types and various signaling pathways control the metastatic process. Metastasis is a multistep process. Many signaling pathways and molecules are involved in metastasis. Increasing knowledge about the mechanism of metastasis can help in finding the promising targets of cancer therapy.

## Introduction

Metastasis means the dissemination of the cancer cells from one organ to another which is not directly connected to the primary site. Metastasis has a crucial role in the prognosis of cancer patients. In the first stage of metastasis, cancer cells detach from the primary tumor and disseminate in the tissue. Then, cancer cells enter the vascular or lymphatic channels (1-3). The survival of the circulating tumor cells (CTCs) inside the lymphatic or blood channels is critical for metastasis to establish a micro-metastasis. Finally, cancer cells extravasate through the vessel wall and proliferate at the secondary site. For each step, a lot of signaling pathways are involved. A few theories have been proposed to explain the mechanism of metastasis. (a) The “organ selection concept” suggests that the growth factors establish a successful metastasis in the metastatic site (4, 5). (b) The “adhesion theory” proposes that the tissue specific adhesion molecules which are expressed on endothelial cells of recepient organs anchor the migrating cancer cells, therefore, provide a premetastatic niche. (c) The “chemo-attraction theory” explains that the cancer cells express chemokine receptors. (d) Paget in 1889 first reported the theory of “seed” for metastatic tumor cells and of “soil” for the secondary site. According to this concept, the organ distribution is determined by the site and histopathological type of the primary tumor. (e) Recently, premetastatic niche has been indicated to explain metastasis. This concept proposes that prior to co-localization; the primary tumor induces the microenvironment of secondary site by CTCs. In the next step a metastatic niche is generated to support disseminated tumor cells (DTCs) and localize them to develop a metastasis. (f) The last one is a new theory that describes a bidirectional relationship between the primary and secondary sites. According to this theory, the surviving cancer cells in the metastatic tumor can return to the primary site to promote the primary tumor progression (6, 7).

Metastasis to a specific organ is determined by some factors such as circulation patterns. Efficient and direct blood flow can explain the probability of metastasis to the specific organs like hepatic metastasis in patients with colon cancer which receive direct blood flow from the primary site (8). The other factor is vascular permeability which significantly promotes extravasation at the metastatic site (9). At the present time, understanding of molecular mechanisms of metastasis remains incomplete. This review focuses on recent advancement in molecular pathways of metastasis. Many molecular pathways and signals are involved in cancer metastasis, but only some of them were discussed in this review article.

## Materials and Methods

In this work, the related articles in the limited period of time, 2000–mid -2018 were reviewed, through search in PubMed, Google Scholar and Scopus database. The articles published in the last two decades related to the biology of cancer metastasis were selected and the most important factors were discussed.

## Results

***Cancer stem cells***

In the last decades, cancer stem cells (CSCs) also known as tumor initiating cells (TICs) have been identified to explain tumor growth, tumor recurrence and metastasis and tumor chemo-resistance (10). It has been suggested that cancer tissue contains a small subset of tumor cells involving cancer development. These cells are not only able to reproduce the original phenotype but also capable of self-renewal (11-13). Self-renewal is crucial to maintain the cell stemness, which is important in tumor cell proliferation and differentiation. Asymmetric cell division of CSCs helps the cells to have a low proliferation rate with long-lived properties. These characters enhance the widely spread of CSCs and resistant to cytotoxic conditions. Chemo-resistance is the major issue of treatment failure of cancers resulting in cancer recurrence and is also associated with acquisition of more aggressive form of cancer cells and/or metastatic type (14). Besides, CSCs explain intra-tumoral heterogeneity (15). CSCs may have a great impact on metastasis as they migrate and attach to a new location (16). Inactivated apoptosis signaling pathways help CSCs to survive, possess self-renewal properties and elude cytotoxicity of most anticancer therapies (17). Regarding the origin of CSCs, there are two theories. One suggests that CSCs originate from normal stem cells or progenitor cells. The other theory indicates that CSCs originate from normal cells which acquire stem cell-like properties via epithelial-mesenchymal transition (EMT) process. In squamous cell carcinoma (SCC), CSCs have two different phenotypes. One phenotype is similar to normal epithelial stem cells related to tissue growth and the other is similar to the migrating CSCs acquiring EMT-associated genes (18). Because of the similarities between physiological stem cells and CSCs, experimental cancer stem cell research faces a lot of difficulties. To overcome these difficulties, cancer stem cell markers are needed but the markers are not specific to cancer stem cells and are found also on normal stem cells. A variety of markers has been suggested to identify CSCs; however, the clinical significance of these markers is not clear.  Nonetheless, previous studies have indicated that CSC surface markers are considered as molecular targeted therapies for different types of cancer (19).  A majority of cell surface proteins and a number of intracellular proteins have also been postulated to function as CSC markers. Immunohistochemistry of primary tumors is proposed as a useful technique to identify CSC markers. The problem arises in the identification of CSC markers involved in metastasis and in therapeutic resistance due to the difficulty of obtaining metastatic or recurrent biopsies. It is necessary to combine markers to get very high specificity which is tumor type–dependent; however, the use of multiple markers significantly decreases the numbers of cells isolated from cancer patients making the diagnosis more difficult (20). Several biomarkers have been identified for CSCs. Among them CD44, CD133, and ALDH1 are the most widely studied. Some epithelial cells and macrophages express the CD44 variant (CD44v) isoforms at various stages of development. In normal tissues, CD44 serves to activate lymphocytes, release of cytokines and regulate hyaluronic metabolism. Hence, it involves in wound healing, and keratinocyte proliferation. Besides, CD44 makes non-metastatic cell lines to become more metastatic (20). The degradation of extracellular matrix (ECM) is regulated by matrix metalloproteinases (MMPs) (21). Emerging literature reveals the association of CD44 with MMP-9 in mouse and human tumor cells to enhance the invasion of cancer cells (22). Localization of MMP-9 on the surface of keratinocytes depends on its interaction with CD44 which activates transforming growth factor-β (TGF-β), and is essential for the promotion of tumor invasion and angiogenesis (23). Previous published work has shown that CD44 and MMP-9 co-localize in tumor cells at the invasive front (24). CD44 is also a marker associated with EMT and CSCs in oral cancer and promotes cancer cell aggressiveness by targeting extracellular signal-regulated kinases 1, 2 (ERK1/2) (25). CD44 may control bone metastasis in hematopoietic cancers and solid tumors. The interaction between CD44 and hyaluronan acid may facilitate the entrance of CSCs into bone marrow and their colonization (26).

CD133, a cell surface glycoprotein, is another CSCs marker (27). CD133-positive cells exist in both primary and metastatic tumors; however, CD133-positive cells are more numerous in metastatic sites. Several lines of evidence indicate that elevated expression of CD133 is correlated with increased migration, stemness and tumorigenicity level due to induction of EMT (28). CD133 also contributes to metastatic process in several cancers such as colon cancer, and pancreatic cancer (28, 29). CD133+ stem-like cells promote invasion ability and tumorigenesis compared with CD133- cells (30). CD133+ cells enhance activity of the nuclear factor kappa-light-chain-enhancer of activated B cells (NF-κB) pathway compared with CD133- cells (28). Importantly, in head and neck squamous cell carcinoma (HNSCC), CD44+ CSCs express higher CD133 levels than CD44- cells (31).

Aldehyde dehydrogenase 1 (ALDH1) is also a biomarker of CSCs in cancers and correlates with the migration of cancer cells and poor prognosis (32). ALDH1 is also involved in cell differentiation, metastasis, detoxification and drug resistance through the oxidation of intracellular aldehydes (33).

**Figure 1:**
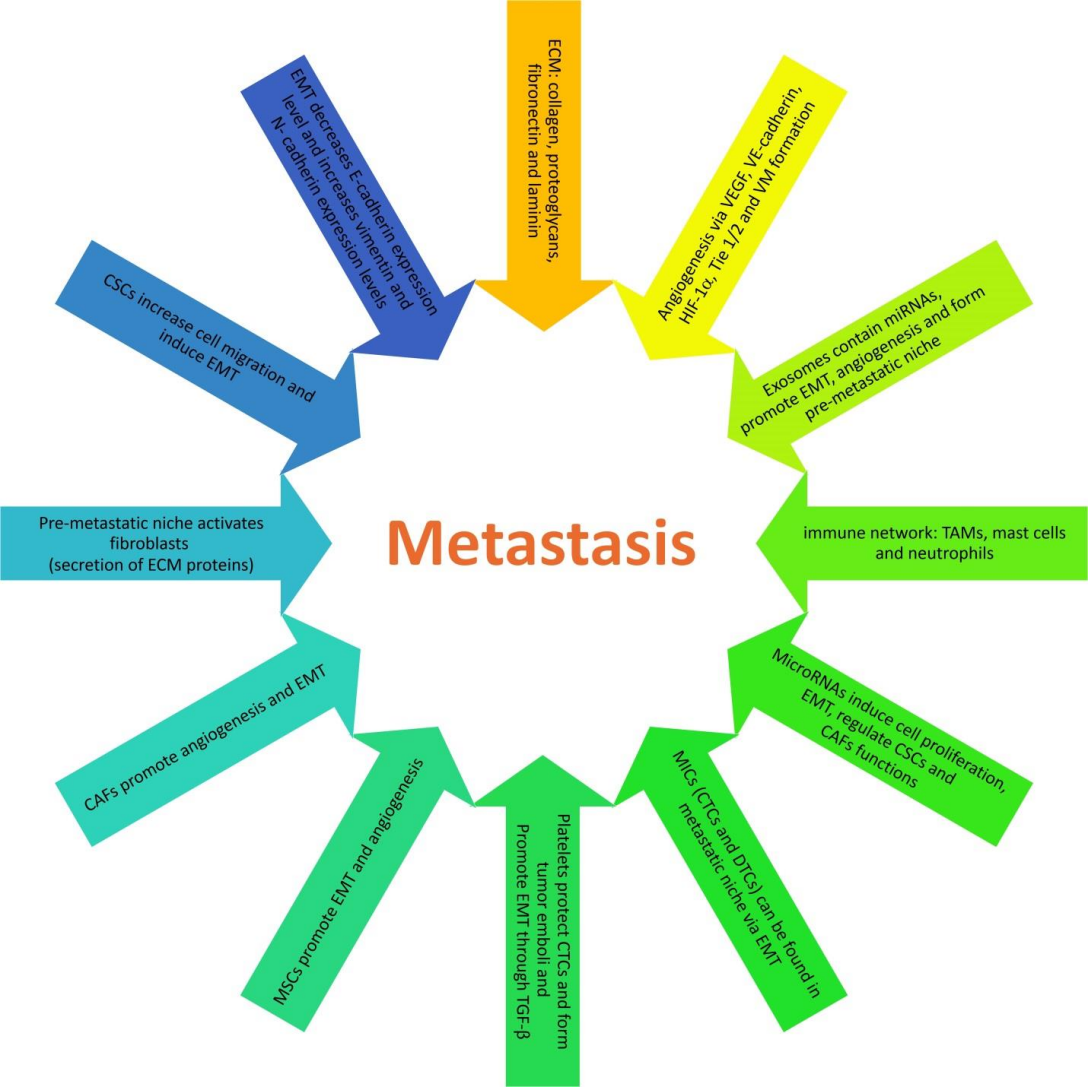
Metastasis is controlled by specific signaling pathways

**Table 1:** A Summary of some tumor suppressor miRNAs and their target genes

**miRNA**	**Cancer type (Reference No.)**	**Involved pathway**	**Target gene**
miR-1	Ovarian cancer (136)	Proliferation and invasion	c-Met
miR-7	Lung cancer (137)	Tumor Growth	TTF-1
miR-7	Hepatocarcinoma (138)	Proliferation and invasion	KLF-4
miR-9-3p	Nasopharynx carcinoma (139)	EMT	FN1,ITGB1 and ITGAV
miR-17-5p	Non-small cell lung cancer (140)	Proliferation and apoptosis	TGFβR2
miR-29a/b/c	Glioma (141)	Invasion	CDC42
miR-30	Lung cancer (142)	EMT	Snail
miR-31	Gastric cancer (143)	Metastasis	RhoA
miR-33a	Melanoma (144)	Angiogenesis	HIF-1α
miR-34a	Bladder cancer (145)	Proliferation and invasion	HNF4G
miR-99a	Cervical carcinoma (146)	Proliferation	TRIB2
miR-100	Esophageal carcinoma (147)	Tumor growth	CXCR7
miR-107-5p	Non-small cell lung cancer (148)	Tumor growth	EGFR
miR-126	Esophageal cancer (149)	Proliferation	VEGF-A
miR-141-3p	Prostate cancer (150)	Bone metastasis	NF-κB
miR-143	Breast cancer (151)	Tumor growth	ERK5,MAP3K7
miR-145-3p	Non-small cell lung cancer (152)	Migration and invasion	PDK1
miR-148a	Renal cell carcinoma (153)	Proliferation and invasion	AKT2
miR-195	Laryngeal carcinoma (154)	Tumor growth and invasion	DCUN1D1
miR-203	Hepatocellular carcinoma (155)	Metastasis	RASAL2
miR-211-5p	Thyroid cancer (156)	Proliferation and invasion	SOX 11
miR-429	Hepatocellular carcinoma (157)	Migration and invasion	CRKL
miR-551b	Gastric cancer (158)	EMT and metastasis	ERBB4

**Table 2:** A summary of some Oncomirs and their target genes

**miRNA**	**Cancer type (Reference No.)**	**Involved in**	**Target gene**
miR-10b	Laryngeal carcinoma (159)	EMT	E-Cadherin
miR-21	Neuroblastoma (160)	Proliferation and apoptosis	PTEN and PDCD4
miR-24	Breast cancer (161)	Proliferation and invasion	SFRP4 and LATS2
miR-93	Hepatocellular carcinoma (162)	EMT and Metastasis	PDCD4
miR-135b	Pancreatic cancer (163)	Metastasis	SFRP4
miR-155	Clear-cell renal-cell carcinoma (164)	Migration and invasion	FOXO3a
miR-181	Non-small cell lung cancer (165)	Chemo-resistance	PI3K/AKT/mTOR
miR-191	Cervical cancer (166)	Proliferation	CDK9, NOTCH2, and RPS6KA3
miR-223	Lung cancer (167)	Invasion	EPB41L3

CD24, a small cell surface protein, is expressed in different cancers such as breast, ovarian, prostate and bladder cancers. As it contributes to cell adhesion and metastasis, it might be a marker in cancer prognosis and diagnosis (34). CD24 cell membrane and intracellular expression in pancreas cancer inhibits the cancer cell invasion and metastasis (35). The other CSCs markers are CD26, CD29, CD166 (36). EMT phenomenon also contributes to the tumorigenicity of CSCs and metastasis.

***EMT and cancer progression***

Epithelial- mesenchymal transition (EMT) was first coined by Elizabeth Hey in 1980s to describe epithelial mesenchymal phenotype changes in chick embryo. EMT and its reverse mesenchymal-epithelial transition (MET) are characteristics of cellular plasticity during embryogenesis and tumor metastasis (37). EMT is characterized by the decreased expression levels of E-cadherin and β-catenin and elevated expression levels of vimentin, fibronectin and N-cadherin (11). In cancers, EMT is a master process by which cancer cells lose their epithelial characteristics to acquire mesenchymal-like properties. Tumor cell migration is a pre-requisite for the metastatic process; hence, EMT is the most critical step to initiate metastasis including metastasis to lymph nodes (38). During EMT phenomenon, cancer cells lose their cell-to-cell junctions and cellular polarity via multiple signaling pathways which increase the motilities and invasive phenotype of them (39). MMPs mediate the cleavage of E-cadherin to increase the tumor cell motility and invasion (40). In addition, EMT has a key role in drug resistance. For example, a previous study has detected high levels of vimentin in adriamycin and vinblastine resistant breast cancer cell lines (41). EMT phenomenon promotes CSCs motility, cancer cell invasion, tumor metastasis and recurrence and drug resistance. Expression of stem cell like markers and formation of tumor spheres by CSCs are enhanced by EMT process. CSCs acquire mesenchymal features by undergoing EMT phenomenon. By doing this, CSCs become resistant to anti-cancer therapies; hence, they can survive and cause cancer recurrence. Besides, CSCs invade to the adjacent stromal tissues, enter the vascular channels, and finally reach the distant organs. In the target organs, CSCs cause MET phenomenon which results in the acquisition of epithelial characteristics. In addition, MET phenomenon increases the cell-to-cell attachment, cancer cells proliferation and differentiation to form metastatic lesions (42). Taken together, EMT induces CSC properties as well as metastatic activities. On the other hand, EMT and CSCs collaborate in invasion capacity of invasive front zone; therefore, targeting the EMT/CSC phenotype can be a therapeutic therapy for the treatment of metastasis and tumor recurrence (43).

***The invasive front area of cancers***

Invasion is the initial step in the metastatic process. The mode of tumor invasion is an important prognostic factor in the cases of presence of lymph node metastasis. Both EMT and MET are essential for cancer progress, as EMT of primary tumor cells is a necessity for motility and invasiveness and MET is important for the final stage of metastasis when the extravasated cancer cells revert to epithelial cells and proliferate as a secondary tumor in the metastatic site (11-13). The interface between the growing primary tumor and the tissue stroma is called” the invasive front”. The invasive front zone is composed of stromal cells such as fibroblasts, myofibroblasts, myeloid progenitor cells, immune cells including tumor-associated macrophages (TAMs) and many blood vessels (44). Signals from these cells promote the cancer cell invasion. At the invasive front, TAMs promote EMT, invasion and cancer stem cell (CSC)-like properties via transforming growth factor beta (TGF-β) (45). Activation of Wnt/β-catenin pathway induces cell motility and invasion. Wnt/β-catenin and RAS-extracellular signal-regulated kinase (ERK) pathways are essential for formation of CSCs and metastasis (46). High level expression of CSCs markers such as CD44 has been detected at invasive front in OSCC and mucoepidermoid carcinoma (MEC) (11, 13). Besides, high expression levels of vimentin, another EMT marker, and vascular endothelial cadherin (VE-cadherin), a tumor angiogenic and progression marker, have been shown at invasive front in OSCC and MEC (12, 13). 

***Role of ECM in cancer progression and its remodeling***

ECM is composed of collagens, elastin, fibronectin, laminins, proteoglycans/glycosaminoglycans and some other glycoproteins and is a non-cellular three-dimensional macromolecular network of tissues. The components bind to each other and to the cell surface receptors to form a network. This network helps the cells to reside in all tissues and organs. These components make up the interstitial matrix and basement membrane (BM). Basement membrane is a specialized ECM which separates the epithelium or endothelium from stroma. Basement membrane is less porous and more compact than the interstitial matrix and is composed of type IV collagen, fibronectin, laminins and some linker proteins such as nidogen and entacin. Interestitial matrix is rich in fibrillar collagen, proteoglycans and some other glycoproteins such as tenacin C and fibronectin. Under pathological conditions, the biomechanical properties of ECM can change which have a great impact on the cell migration. Additionally, ECM is a dynamic environment which is constantly remodeled in different tissues at developmental stages; embryonic and postnatal. Lastly, cell-ECM interactions allow cells and tissues to adapt with the environment (47). Many matrix-degrading enzymes continuously rebuild ECM during normal and pathological conditions. ECM has a critical role in the maintenance of cell and tissue structure and function and organ morphogenesis. Therefore, the deregulation of composition results in several pathological conditions (48). Remodeling of ECM including basement membrane is one of the master switches for cancer invasion, neo-angiogenesis and metastasis (49). Moreover, ECM has a leading role in maintaining of stem cell properties and controls the stem cell differentiation. Stem cells are located in a specific microenvironment, called niche and play a critical role in the regeneration and maintenance of tissues (50). Normal niches are composed of ECM components, endothelial cells and perivascular cells or their progenitors as well as fibroblasts, immune cells, cytokines and growth factors (51). Thus, ECM functions as a reservoir for growth factors by making them unavailable, insoluble and non-functioning. Collagen, fibronectin, and proteoglycans, for example, bind to fibroblast growth factors (FGFs), vascular endothelial growth factor (VEGF), hepatocyte growth factor (HGF), transforming growth factor beta (TGF-β) and bone morphogenetic proteins (BMPs) (50). The lines of evidence indicate that the mediators of cell-ECM interactions such as integrin receptors provide transducing signals and physical links with the cytoskeleton from ECM to the cell protein modification processes including proliferation, migration and survival (52). CSCs need nutrients and signals from the surrounding microenvironment, also called CSCs niche, to achieve a balance between differentiation and self-renewal. In cancers, niche is a specialized local microenvironment including cancer-associated fibroblasts (CAFs), immune cells, non-CSC cancer cells, blood and lymphatic vessels, ECM, growth factors and cytokines. The expression and secretion of cytokines and growth factors regulate the cell-cell interactions between CSCs and stroma (12, 53). CSCs niche enhances CSCs differentiation and inhibits apoptosis. Moreover, niche may promote the accumulation of gene mutations by stem cells which lead to their malignant transformation into cancer cells (51). ECM promotes tumor progression by providing proliferative signals and disseminating tumor cells (54). Cancer cells release chemo-attractants from distant tissues; thus, interactions between tumor cells and stroma promote cancer development and metastasis (9). In the early stage of cancer metastasis, cancer cells degrade the epithelial basement membrane and come into contact with tumor stroma. Tumor cells invade the local stroma either in clusters/sheets (collective model) or as a single cell and enter the blood vessels (44). MMPs, the proteolytic enzymes, are necessary to degrade ECM (21). For example, in retinoblastoma, MMP2 and MMP9 degrade type IV collagen to facilitate invasion. MMP9 also promotes angiogenesis in cancers (55). The over-expression of MMP9 and MMP2 has been observed in head and neck squamous cell carcinomas patients presenting with lymph node involvement (56, 57). Bone metastasis is a frequent event in different cancers. Some factors which release growth factors in ECM promote bone degradation which release growth factors in ECM. Bone destruction enhances metastatic outgrowth (49). Metastasis to the liver is another predicting factor. Collagen IV is the main component of ECM within liver. Binding of collagen-IV to integrins (in particular integrin-α2) has been implicated within hepatic metastasis. Collagen-IV rescues the cancer cells from anoikis within the liver (58). 

***Anoikis apoptotic process***

Anoikis is a Greek word and means loss of “home” or “homelessness”. It is a particular apoptotic process in response to loss of cell adhesion to ECM. Separation of a normal epithelial cell from its ECM results in anoikis. There are two apoptotic pathways; the mitochondrial (intrinsic) pathway and cell death receptor (extrinsic) pathway (59). Anoikis is essential to maintain tissue homeostasis by eliminating displaced endothelial / epithelial cells and therefore, preventing those cells from inappropriate seeding. Resistance to anoikis is a hallmark of EMT phenomenon and is a pre-requisite for metastasis (52). Also, resistance to anoikis increases the number of circulating tumor cells (CTCs) to facilitate recurrence and metastasis (60). A previous study has revealed that the heavily glycosylated mucin protein (MUC1) which over-express in all types of epithelial cancer cells can prevent the initiation of anoikis in response to loss of cell adhesion (61). Besides, activation of insulin receptor or insulin-like growth factor receptors has a key role in cancer cell resistance to anoikis. The exact role of p53 in anoikis has not been demonstrated; moreover, a protective role of the p53-via induction of ECM and expression of integrin gene has been shown. Another factor related to anoikis is E-cadherin. Knockdown of E-cadherin enhances cancer cell resistance to anoikis which results in EMT phenomenon. Both the TGF-β and Wnt pathways induce EMT which drives anoikis resistance (62). In contrary, Bcl-2 inhibitor of transcription 1 (Bit1) promotes apoptosis. After loss of cell attachment, Bit1 is secreted to the cytosol and interacts with the transcriptional regulator amino terminal enhancer of split (AES) to induce a caspase-independent form of apoptosis. Additionally, Bit1 inhibits EMT which has a great impact on tumor progression and aggressiveness; thus, it is considered as a tumor suppressor. Down–regulation of Bit1 in cancers such as lung adenocarcinoma enhances anoikis anchorage-independent growth and resistance, which results in tumorigenicity and metastasis (63). Platelets induce anoikis resistance, and promote metastasis via their association with extravasating tumor cells or detached. Cancer cells activate platelets via secretion of some factors such as adenosine 5′-diphosphate (ADP) which in turn leads to release the pro-angiogenic and pro-tumorigenic factors (64). These findings provide new insight into the molecular mechanism of anoikis regulation in cancer progression and metastasis. 

***Pre-metastatic niche***

In 2005, Kaplan and colleagues first introduced the concept of pre-metastatic niche. They demonstrated that bone marrow-derived hematopoietic progenitor cells can express vascular endothelial growth factor receptor 1 (VEGFR1) which accumulates in the pre-metastatic lung tissue before tumor cell arrival (65). The environment at the distant target organ called pre- metastatic niche and is induced by factors derived from the primary site. A pre-metastatic niche is a supportive environment for cancer growth in the host tissue before spread of cancer cells. The formation of the pre-metastatic niche is a critical step towards metastasis (66). Host microenvironment is modified by primary tumor via secretion of some factors such as tumor cell-secreted factors and exosomes, host cell (fibroblast) alternations and non-resident cell (haematopoietic progenitor cells) recruitment (67). The pre-metastatic niche contains activated fibroblasts which secrete high levels of ECM proteins like type I collagen (68). In addition, tumor cells-host ECM interactions result in increasing capillary permeability and clot formation which promote metastasis (67, 69). In other words, for successful metastasis, tumor cells not only have to get specific genetic properties but also need to prepare the local microenvironment at the distant recipient organs. Metastatic cancers have preferences for specific target organs and specific microenvironments. For instance, metastatic breast cancer cells often migrate to the pre-metastatic niches located at the lungs, liver, brain, bone, and lymph nodes. These findings suggest that each tissue has specific characteristics that promote tumor cell homing, adhesion, and growth (7, 70). Hence, the distribution of distant metastases to particular organs is not a random process (metastatic organotropism)(69).

***Angiogenesis in cancer***

In 1971, Folkman first proposed a theory regarding tumor angiogenesis. According to his hypothesis, a tumor produces its own new blood supply from existing blood vessels. Angiogenesis has an essential role in tumor growth and metastasis. Accumulating lines of evidence have indicated that some cancer blood vessels are not lined by endothelial cells (71). Maniotis *et al. *first found that the aggressive melanoma cells form vascular-like channels which function as tumor blood vessels to supply nutrition. This phenomenon was called “vasculogenic mimicry” (VM) (72). VM formation enhances tumor growth and cancer metastasis (71). In the early stage of VM formation, the vascular channels are lined by tumor cells and VM is the main source of blood supply. Over time, endothelial cells differentiate and proliferate to contribute to vessel formation. In this stage, the vessels are lined by both tumor cells and endothelial cells, called” mosaic pattern”. Lastly, endothelial lined vessels replace VM and mosaic vessels (73). VM formation demonstrates the ability of tumor cells to differentiate into endothelial-like cells to form blood channels independent of the endothelial cells. The transformed endothelial-like cells produce matrix proteins such as collagens IV and VI, laminin, proteoglycans and heparan sulfate which help to form tubular networks. Histologically, VM channels are lined by tumor cells with a basement membrane in the external wall where there is not any endothelial cells on the inner wall (11, 12, 74).

The plasticity and heterogeneity of cancer cells promote the acquisition of endothelial cell markers such as vascular endothelial growth factor receptor 2 (VEGFR2), CD31 and VE-Cadherin (CD144). Hypoxia is an important factor for VM formation which up-regulates VE-cadherin expression (75). Angiogenesis is a master switch in the development of metastasis not only because of supplying the nutrition but also because of providing some pathways for cancer cells which travel to other organs to form new tumors. Production of VEGF is the main signaling pathway for angiogenesis (76, 77). Tumor cells, reactive stromal cells and infiltrating inflammatory cells are the sources of VEGF (78). Besides, over-expression of hypoxia-induced factor-1 α (HIF-1α) induces EMT and metastasis in cancers such as head and neck cancer. Co-expression of HIF-1α, Snail and Twist is associated with metastasis and poor prognosis in human head and neck cancers (79). The Tie receptors 1 and 2 (Tie1/2) have essential roles in embryonic angiogenesis as well as tumorigenesis. Elevated expression of Tie1 has been detected in different types of cancer and has a negative correlation with clinical outcome. Besides, Tie1-positive cells show cancer stemness properties (80). Recent findings indicate that CSCs initiate tumor neovascularization. CSCs can differentiate to endothelial cells and vascular smooth muscle-like cells. In addition, CSCs form VM channels. For instance, a previous published work on glioma demonstrated that glioma stem cells expressed both neoplastic and endothelial markers and VM positive tumor tissues showed elevated expression levels of some genes associated with CSCs (81). Under hypoxic conditions, CSCs express hypoxia-inducible factors (HIFs) which are controled by TGF-β. HIFs are the primary factors for promoting angiogenesis through induction of VEGF. Both endothelial cells and CSCs produce VEGF. VEGF-A can recruit monocytes and macrophages (51). In addition to ECM and angiogenesis, several different cell types are involved in cancer metastasis.

***Mesenchymal stem cells***

Mesenchymal stem cells (MSCs) are multipotent mesenchymal stromal cells which first described by Friedenstein *et al.* as fibroblast-like cells in the bone marrow (82). Some other tissues like placenta, and adipose tissue also contain MSCs (83). MSCs allow a cellular population to generate diverse cell types and can be characterized by specific cell surface markers. More than 95% of the cell population expresses CD105, CD73, CD44 and CD90 (84, 85). Due to proliferation and differentiation potential of MSCs, they are novel opportunities for some clinical applications, such as cell therapy, cancer gene therapy, treatment of graft versus host disease and regenerative medicine. Besides, MSCs are nearly unidentifiable by immune system which helps them to migrate through the circulation. In addition, because of low immunogenicity of MSCs they are novel therapeutic approaches even without HLA matching (86). The unique characteristic of MSCs is the ability to migrate to sites of inflammation, tissue injury and cancerous tissues (87). MSCs also suppress immune response via inhibition of T-cell proliferation, dendritic cell maturation and natural killer (NK)/B-cell activation (88). Cancer cells provoke a chronic inflammatory response within the tumor microenvironment via producing inflammatory chemokines and growth factors. Some of chemokines associated with angiogenesis and tumor progression are epidermal growth factor (EGF), fibroblast growth factor (FGF), granulocyte colony-stimulating factor (G-CSF), granulocyte–macrophage colony-stimulating factor (GM-SCF),vascular endothelial growth factor-A (VEGF-A), platelet-derived growth factor (PDGF), angiopoietin-1, urokinase-type plasminogen activator (uPA), IL-6, IL-8 and TGF-β1 (86). Within the tumor microenvironment, MSCs have this ability to differentiate into cancer associated fibroblasts (CAFs) to support tumor progression (89). MSCs participate in several crucial steps of invasion and metastasis, such as EMT phenomenon (90). Cancers contain a number of factors for activating and recruiting of MSCs. In turn, MSCs modulate biological properties of tumor cells directly by EMT phenomenon. Migration of MSCs toward the primary and metastatic tumor microenvironments has been indicated in some cancer types such as skin cancer and lung cancer (91). In cancer microenvironment, MSCs also induce angiogenesis and resistance to drugs (92). In general, MSCs enhance cancer cell proliferation, angiogenesis and metastasis. 

***Cancer- associated fibroblasts***

Cancer-associated fibroblasts (CAFs) are spindle shaped cells which morphologically look like myofibroblasts and are one of the most abundant cell types in the stroma (93). A previous study has indicated that bone marrow derived stromal cells and MSCs are the major sources of CAFs (94). Accumulated documents reported a cross talk between cancer cells and CAFs. In several cancers, the presence of CAFs in the stroma is associated with poor prognosis and increased risk of metastasis (95). CAFs promote tumor proliferation, invasion, and metastasis through producing several factors including cytokines such as uPA and growth factors which cleaves MMPs to induce ECM degradation and to promote angiogenesis and EMT (93). CAFs are involved in tumor cell proliferation via different mechanisms, for instance, in gastric cancer; CAFs target PTEN through the up-regulation of microRNA106b (96). Besides, CAFs improve the ability of cancer cells to invade and metastasize via EMT phenomenon (94) and the secretion of angiogenic factors such as VEGF and angiopoietin. On the other hand, CAFs promote the infiltration of immune cells in cancer tissues by producing inflammatory mediators such as chemokine (97). CD44 is expressed in CAFs and enhances the interactions between cancer cells and CAFs which may suggest the contribution of CD44 in tumorigenicity, stemness and drug resistance (11, 13, 98). CAFs are mainly located at the tumor periphery (93). CSC-like cells make-up a heterogeneous population of cells surrounded by myofibroblast-like cells. It is hypothesized that CSCs might be the source of the CAFs and support tumor maintenance and survival. In turn, CAFs support CSC self-renewal (99). Furthermore, in prostate cancer, cancer-associated fibroblasts (CAFs) induce EMT via the secretion of MMPs (51).

***Metastasis initiating cells, circulating tumor cells, and Circulating tumor microemboli***

Metastasis-initiating cells (MICs) are cancer cells with the ability of seeding in the secondary organs. The tumor-initiating cells (TICs) are the primary tumor counterparts of MICs and both increase the cancer cell plasticity and stemness. However, MICs need to get additional capabilities which enable them to survive the function and metastatic cascade as TICs in distant target organ microenvironment (100). MICs might represent a subpopulation of CSCs. MICs might be early- stage disseminating CSCs or might obtain from late-stage disseminating CSC clones (101). Metastasis results from the successful circulation of primary cancer cells into a distant organ; hence, it is logical to expect to find MICs among circulating tumor cells (CTCs) as well as disseminated tumor cells (DTCs) in the metastatic niche (102). CTCs are a cell population with heterogeneity in number, size, and clustering status as well as the dissemination pathways. CTCs are at a very low concentration especially in early tumor stages and are associated with poor prognosis. Detection of CTCs prior and during therapy has a great impact on cancer therapy outcome (92). CTCs infiltrate the circulation by the acquisition of EMT characteristics and survive within the circulation (103). However, not all CTCs and DTCs are capable of forming micro- or macro-metastasis, as most of the cells persist within the metastatic tissue but do not survive the oxygen tension changes and shear stresses (20). In the circulatory system, CTCs form cell clusters or circulating tumor microemboli (CTM). CTM is composed of 2 to 50 cells. In some cases, it is made up of more than 100 cells. CTM has a high capacity of metastasis compared to single cell. Furthermore, the contribution of CTM in metastatic process has been reported. Notably, discovering CTM, even a single one in the cancer patient blood significantly correlates with worse prognosis and decreased disease- free survival rate. In addition to cancer cells, CTMs contain some other cell types such as platelets, endothelial cells, fibroblasts, leukocytes and pericytes. Due to rapid entrapment in small capillaries, CTMs have a short half-life. Nevertheless, it has been shown that CTMs containing less than 20 cells form a single file pattern to traverse small blood channels. A previous study on melanoma indicated that cancer cells produce VEGF-A which forms micronodules of cancer cells. In the next phase, VEGF-A promotes vascular dilation to facilitate the intravasation of micronodules. A growing body of evidence indicates that dissemination of cancer cells is an early event in cancer development. Although, to get metastatic ability, they need further evolution at the metastatic site. EMT phenomenon might be a key mechanism which induces mesenchymal phenotypes of the cells within a CTM to promote metastasis. Higher expression level of plakoglobin, a cell-cell adhesion molecule, has been demonstrated in CTM compared to single CTC. Recent publications have reported that the cells within CTMs are negative for proliferation marker Ki67 which may indicate that these cells are chemo-resistant and may reflect the necessity of new drug development (104). 

***The role of platelets in the survival of CTCs***

Some cancer cells secrete platelet-activating mediators such as thrombin , ADP and thromboxane A2 (TXA2) (105). Metastatic cancer cells induce the aggregation of platelets, in turn, platelets contribute to survival of CTCs in the blood stream (106). Autotaxin (ATX) an enzyme released by platelets, enhances cancer cell proliferation, angiogenesis and metastasis (107). Serotonin, another factor released by platelets, activates angiogenesis by enhancing the proliferation of endothelial cells (108). After activation of platelet, its granules release TGF-β1 which promotes EMT phenomenon. Besides, the increased number of platelets is associated with chemo-resistance and poor prognosis (109, 110). Platelets coat CTCs to protect them from shear forces; however, most CTCs die in circulation due to shear stress and/or anoikis (111). Additionally, tumor cells bind to the platelet adhesive proteins such as fibronectin and von Willebrand factor via integrins to form tumor emboli. In the next phase, tumor emboli can anchor to the luminal side of endothelial cells to get a greater chance of entrapment in small vessels which give more time to tumor cells to extravasate. CTCs may reach to the metastatic site even before the appearance of clinical features; thus, the presence of CTCs is associated with a poor prognosis (112).

***Tumor immune network***

Inflammatory response is another characteristic of cancer. Different immune cells have different impacts on tumors. Some immune cells act as promotor and some as antitumor. Besides, they play crucial roles in therapeutic resistance (113). Different types of immune cell are involved in cancers including macrophages, dendritic cells, mast cells, B cells, effector T cells (T-helper cells and cytotoxic T cells) and natural killer (NK) cells (114). Chronically activated leukocytes produce some mitogenic growth factors. For example, EGF, TGF-β, tumor necrosis factor alpha (TNF-α), fibroblast growth factor, interleukins (ILs) and chemokines. Additionally, granulocytes, monocytes, macrophages and mast cells secrete proteolytic enzymes to modify the structure and function of ECM. Moreover, in chronic inflammatory responses, leukocytes enhance angiogenesis and migration of cancer cells (115).

Tumor-associated macrophages (TAMs) might be the most abundant inflammatory cells which mainly aggregate within the tumor tissues rather than in peri-tumoral tissues. The number, phenotype and distribution pattern of TAMs influence patient outcomes (116). Interactions between cancer cells and macrophages play a critical role in regulation of ECM and immune surveillance of cancer cells. Previous studies have revealed that macrophages are heterogeneous cells which may explain the plasticity of these cells. High plasticity helps them to adapt to complex environments such as cancer (117). They are divided into M1 and M2 macrophages. They have paradoxical properties, for example, M1 type macrophages kill pathogens and enhance the activation of cytotoxic T lymphocytes (CTLs). Thus, they are correlated to more favourable prognosis and function as anti-tumor. In contrast, M2 type macrophages stimulate a CD4+ and regulatory T-cell response to promote angiogenesis and tissue remodeling. M2 type macrophages activate secretion of interleukin 6 (IL-6), TGF-β and interleukin 10 (IL-10), and VEGF to enhance tumor growth which are associated with a worse prognosis (114). M1 macrophages express type-1 cell-attracting chemokines such as C-X-C motif chemokine 10 (CXCL10) and Chemokine (C-X-C motif) ligand 9 (CXCL9) but M2 macrophages express the Chemokine (C-C motif) ligand 17 (CCL17), C-C motif chemokine 22 (CCL22) and Chemokine (C-C motif) ligand 24 (CCL24) (118). In glioblastoma, CSCs produce cytokines via activation of the STAT3 pathway to recruit and polarize macrophages to become M2-like cells, in turn, M2- like cells serve as a CSC niche to enhance CSC growth (119). 

CD4+ T helper cells have a key role in the inflammatory processes in cancers via secretion of a set of cytokines. CD4+ T cell contribute to tumorigenesis through different mechanisms. For example, regulatory T cells (Tregs), an immunosuppressive subset of TH cells, prevent cytotoxic functions of CD8+ T cells to avoid rejection of tumor. TH cells also contribute to angiogenesis and recruitment of myeloid cells especially neutrophils. In breast cancer TH2 cells increase the risk of metastasis to sentinel lymph nodes by the expression of GATA binding protein 3 (GATA-3) (113).

Mast cells, another type of inflammatory cells, are present within the tumor and peri-tumoral microenvironment, termed tumor –associated mast cells (TAMCs) (120). The role of mast cells in tumor progression is still controversial. For example, a previous study has found that decreased number of mast cell in peri-tumoral stroma of deeply invasive melanoma is associated with poor prognosis; however, mast cell density is not a prognostic factor for superficially invasive melanoma. These results indicate that the role of mast cells in melanoma depends on the phase of tumor progression (120, 121). Another study has indicated that mast cells promote the growth of cancer in the initial stages not later stages of prostate cancer by producing MMP-9 in ECM (122). Mast cells produce several pro-angiogenic factors such as VEGF-A, -B, and FGF-2 as well as lymphangiogenic factors including VEGF-C and –D (120). In oral squamous cell carcinoma (OSCC) increased microvessel density is associated with increased mast cell density and is correlated to poor prognosis (123-125). Besides, mast cells induce EMT and stem cell features in human cancer via the synthesis of CXCL8 (IL-8), a member of the chemokine family (120). 

Neutrophils also play a critical role in host inflammatory responses. Neutrophils regulate cancer development and metastatic processes. These cells can display either pro- or anti-tumor characteristics. Pro-tumor neutrophils enhance angiogenesis through producing pro-angiogenic factors. Endothelial cells recruit the immune cells such as neutrophils. Hence, activation of endothelial cells has a great impact on the regulation of metastasis. Besides, neutrophils have immunosuppressive properties and can limit anti-tumor immune responses. On the other hand, neutrophils have anti-tumor properties, such as ability to kill tumor cells. In addition, neutrophils stimulate anti-tumor responses leading to the activation of T cells and tumor rejection (66). Several lines of evidence point to the role of the neutrophils as the main population of cells in pre-metastatic niche. Neutrophils promote metastasis through extracellular matrix remodeling, angiogenesis, proliferation of cancer cells, promotion of invasion, and induction of inflammation at secondary sites. Granulocyte colony-stimulating factor (G-CSF) organizes neutrophils and simplifies their homing at distant target organs prior to the arrival of cancer cells (66). Besides, the aforementioned factors and different cell types some other factors such as exosomes, miRNAs and circRNAs are involved in cancer progression and metastasis.

***Exosomes***

Exosomes are small microvesicles (sized about 50–100 nm) obtain from cellular endocytosis (via multivesicular endosome pathway and reverse inward budding) and contain common proteins, such as membrane trafficking proteins (Rab proteins, ARF GTPases, and annexins), tetraspanins (CD9, CD63, and CD81) and cytoskeletal proteins. Besides, exosomes contain mRNAs, miRNAs and specific lipids encapsulated in a cholesterol-rich phospholipid membrane while playing critical roles in long-distance intercellular communications (126, 127). Exosomes are the smallest extracellular vesicles secreted by almost every cell type including mast cells, T lymphocytes, dendritic cells, platelets, epithelial cells, and neurons. They alter the functions of recipient cells by transferring bioactive molecules (127) and contribute to some interactions between endothelial cells, infiltrating immune cells, MSCs, and cancer cells. (128). Exosomes are released by cancer cells in the surrounding peripheral circulation and microenvironment. Exosomes derived from CAFs collaborate with cancer cells to optimize the tumor microenvironment (126). In tumor microenvironment, exosomes can modify recipient cell phenotypes through the exchange of genetic information (129). TAMs and MSCs also produce exosomes in various cell types to promote cancer cell growth and metastasis (127). Besides, the shedding of exosomes by cancer cells under normoxic and hypoxic conditions may promote EMT phenomenon, angiogenesis, and formation of pre-metastatic niches, metastases at distant tissues and organs, and treatment resistance (130). Exosomes have been reported to transfer the properties of chemo-resistance among different types of cancer cells and inhibits apoptosis (131). Exosomes also enhance the metastatic potential of primary tumors through MET phenomenon (132). Exosomes present in all body fluids such as urine, amniotic fluid, cerebrospinal fluid, plasma, synovial fluid and saliva (133).

***MicroRNAs and circRNAs regulate cancers***

MicroRNAs (miRNAs) are endogenous small non-coding RNAs. They can regulate gene expression in the post–transcriptional stage by interacting with the 3’ untranslated region (3’ UTR) of the target mRNA. MiRNAs play crucial roles in different cancers including proliferation, differentiation, apoptosis, survival, motility, invasion and metastasis, and morphogenesis. It has been shown that tmiRNAs can be used as novel molecular biomarkers for cancer diagnosis. Regarding the role of miRNAs in cancers, they are classified as two main groups; tumor suppressor miRNAs and oncogene miRNAs (oncomirs)(134). Altered miRNA expression depends on its role as a tumor suppressor or oncogene. Additionally, metastamir, a specialized family of miRNAs, has pro or anti-metastatic effects (134, 135). Different miRNAs have been investigated in a variety of cancers. For each miRNA, several functional roles and target genes have been indicated. Tables 1 and 2 summarize some of these results.

MiRNAs are the major component of exosomes. Transportation of miRNAs by exosomes makes them resistant to degradation by extracellular ribonucleases. Therefore, miRNAs can be transported to different cell types or to the pre-metastatic niche which enable them to influence the gene expression of target cells. Accumulative evidence indicates that miRNAs may regulate CSCs functions and characteristics by controlling the self-renewal and differentiation of embryonic stem cells (168). MicroRNA alterations correlate to the initiation and progression of cancer cell proliferation or inhibition of tumorigenesis. For example, miR-155 is up-regulated in oral cancer and its dys-regulation is associated with the low level of cell division cycle 73 (CDC73), a tumor suppressor gene; therefore, it promotes cancer cell proliferation (169). A previous study on oral squamous cell carcinoma cells indicated that transfecting with miR-125b or miR-100 significantly decreased cell proliferation; however, co-transfection had a greater impact on proliferation than individual transfection (170). However, miR-551b, an oncogenic miRNA, promotes proliferation and invasion in ovarian cancer cells (171). MiRNAs also contribute to cancer development. Previous studies have indicated that dys-regulation of miRNAs plays a crucial role in the progression of oral precancerous lesion from dysplasia to OSCC. For instance, miR-31 negatively controls oral leukoplakia progression through the regulation of fibroblast growth factor 3 (FGF3) (134). In addition, in oral dysplasia, up-regulation of miR-21, miR-181b, and miR-345 is associated with lesion severity (172). The expression level of miR-126 is conversely related to VEGF-A protein in cancers such as oral cancer, esophageal cancer and gastric cancer (149, 173, 174). The miR-17-92 cluster is positively associated with angiogenesis in some cancers; however, individual components of the miR-17-92 cluster show the opposite effects on angiogenesis (134). Over-expression of miR-210 increases HIF-1α expression level and promotes EMT (175). Over-expression or sub-expression of miRNAs in cancers has the ability to activate or block the target mRNAs. Accumulating evidence shows that exosomes can fuse with the target cell membrane to carry miRNAs. For example, MSC-derived exosomes carry pro-angiogenesis miRNAs and transfer these miRNAs to endothelial cells to promote angiogenesis (176). miR-221 is highly expressed in exosomes secreted by MSCs (177). Finally, some miRNAs are involved in CAFs function. Among them, miR-31 and miR-214, are down-regulated and miR-155 is up-regulated in normal fibroblasts to convert the fibroblasts into a CAF-like phenotype (178). 

Recently, circular RNAs (circRNAs), 100 bp to 4 kb in size, have been demonstrated in different cancers. They are classified as three types: intronic type, exonic type and exonic-intronic type. Exonic circRNAs are mostly found in cell cytoplasm, but the other two types have been shown in cell nucleus. Recent studies indicate that circRNAs can block the binding of miRNAs to the 3’ UTR of a specific gene; hence, can regulate the gene expression (179). Circulating miRNAs may play a critical role in the diagnosis of various types of cancer. For example, elevated serum expression levels of miR-20a, miR-27a and miR-423-5p are recognized as biomarkers for gastric cancer (180). Figure 1 summarizes the specific signaling pathways that control metastasis.

## Discussion

Metastasis means the dissemination of the cancer cells from one organ to another which is not directly connected to the primary site and has a crucial role in the prognosis of cancer patients. Metastasis is critical factor to predict survival in patients with advanced cancer and prognosis determines the treatment plan. Metastasis is the main cause of cancer-related death around the world. The field of molecular pathology and new diagnostic methods has rapidly grown in the last decade. According to the recent genetic and molecular findings, a successful metastasis requires both the accumulation of mutations and acquiring more invasive phenotypes and establishment of a pre-metastatic niche (67). Due to high death rate from cancer around the world, it is worth to find therapeutic strategies to eradicate the metastatic process. A variety of therapeutic approaches have been tested for the cancer treatment. 

The presence of CSCs explains several biologic behaviors of cancers such as tumor growth, tumor recurrence and metastasis and tumor chemo-resistance. Inability to eradicate CSCs may be one of the most important reasons of therapy failure. Hence, CSC surface markers are the promising targets for cancer therapy. A growing body of studies demonstrates that the conjugation of an anti CSC surface marker to a cytotoxic drug is able to inhibit cancer cell growth (181). EMT phenomenon starts the initial metastatic step, local tumor cell invasion and intravasation into the blood channels. Besides, EMT has an essential role in drug resistance. Blocking the EMT- related signaling pathways controls tumor, growth and metastasis. Another important factor for metastasis is ECM which promotes tumor progression by providing several factors for dissemination of cancer cells (54) and pre-metastatic niche construction, a promising therapeutic target (67). Consequently, cancer cells release chemo-attractants from distant tissues to enhance metastasis (9). Different cell types such as MSCs, CAFs, and immune cells are novel therapeutic targets as well. MSCs have this ability to gather in the tumor microenvironment; then, MSC-secreted exosomes should be investigated for therapeutic applications (128). Angiogenesis is a crucial for cancer cell growth; therefore, angiogenic factors like VEGF are also promising therapeutic targets (77). CTCs express some surface markers such as EpCAM and different subtypes of cytokeratin (CK) (182). These markers can be used as minimally invasive biomarkers for early diagnosis, prognosis and monitoring of recurrence. Exosome also can be used as a biomarker. It is suggested that specific exosomal miRNAs serve as indicator of the metastatic process. For example, increased expression of serum exosomal miR-203 in cases of colorectal cancer indicates poor prognosis and liver metastasis. Exosomes are shed into biological fluids; hence, can be used as noninvasive tool for diagnosis and follow-up monitoring of cancer patients. CD9, CD63, CD81, tubulin, actin and CD82 are the common biomarkers of most exosomes (183). Moreover, tumor cell–derived exosomes express stem cell–like markers including CD44, CD133 and/or CD105 (131). Hence, exosomes are promising cancer targets. In addition to different intracellular miRNAs, several extracellular/circular miRNAs have been demonstrated outside the cells including body fluids. These miRNAs are considered as biomarkers for several pathophysiological conditions (184). In a metastatic carcinoma, the miRNA expression profile has been demonstrated to be different from a non-metastatic tumor. Therefore, interference with the expression level of miRNAs has an effect on cancer prognosis. These findings provide a therapeutic use for miRNAs (134). Besides, some circRNAs present in human body fluids. Therefore, they may newly found clinical biomarkers in cancers.

## Conclusion

Metastasis is a multistep process. Many signaling pathways and molecules are involved in metastasis. Most of the involved factors can be easily detected due to the presence of reliable detection techniques. However, many other novel and valid techniques are needed to predict target genes and it is still a long a way before these progression can be used as routine clinical practice. Increasing knowledge about the mechanism of metastasis can help in finding the promising targets of cancer therapy (185, 186). Further investigation is necessary regarding the regulatory mechanisms of cancers. 
